# Artificial-intelligence-enhanced synthetic thick slabs versus standard slices in digital breast tomosynthesis

**DOI:** 10.1259/bjr.20220967

**Published:** 2023-04-17

**Authors:** Stephanie Tina Sauer, Sara Aniki Christner, Philipp Josef Kuhl, Andreas Steven Kunz, Henner Huflage, Karsten Sebastian Luetkens, Tanja Schlaiß, Thorsten Alexander Bley, Jan-Peter Grunz

**Affiliations:** 1 Department of Diagnostic and Interventional Radiology, University Hospital Würzburg, OberdürrbacherStraße, Würzburg, Germany; 2 Department of Obstetrics and Gynaecology, University Hospital Würzburg, Josef-Schneider-Straße , Würzburg, Germany

## Abstract

**Objectives:**

Digital breast tomosynthesis (DBT) can provide additional information over mammography, albeit at the cost of prolonged reading time. This study retrospectively investigated the impact of reading enhanced synthetic 6 mm slabs instead of standard 1 mm slices on interpretation time and readers performance in a diagnostic assessment centre.

**Methods:**

Three radiologists (R1-3; 6/4/2 years of breast imaging experience) reviewed 111 diagnostic DBT examinations. Two datasets were interpreted independently for each patient, with one set containing artificial-intelligence-enhanced synthetic 6 mm slabs with 3 mm overlap, while the other set comprised standard 1 mm slices. Blinded to histology and follow-up, readers noted individual BIRADS categories and diagnostic confidence while reading time was recorded. Among the 111 examinations, 70 findings were histopathologically correlated including 56 malignancies.

**Results:**

No significant difference was found between BIRADS categories assigned based on 6 mm *vs* 1 mm datasets (*p* ≥ 0.317). Diagnostic accuracy was comparable for 6 mm and 1 mm readings (R1: 87.0% *vs* 87.0%; R2: 86.1% *vs* 87.0%; R3: 80.0% *vs* 84.4%; *p* ≥ 0.125) with high interrater agreement (intraclass correlation coefficient 0.848 *vs* 0.865). One reader reported higher confidence with 1 mm slices (R1: *p* = 0.033). Reading time was substantially shorter when interpreting 6 mm slabs compared to 1 mm slices (R1: 33.5 *vs* 46.2; R2: 49.1 *vs* 64.8; R3: 39.5 *vs* 67.2 sec; all *p* < 0.001).

**Conclusions:**

Artificial-intelligence-enhanced synthetic 6 mm slabs allow for substantial interpretation time reduction in diagnostic DBT without a decrease in reader accuracy.

**Advances in knowledge:**

A simplified slab-only protocol instead of 1 mm slices may offset the higher reading time without a loss of diagnosis-relevant image information in first and second readings. Further evaluations are required regarding workflow implications, particularly in screening settings.

## Introduction

Digital breast tomosynthesis (DBT) constitutes a widely adopted technique for superior visualisation of pathologies in mammography, which in standard projections are often obscured due to superimposed parenchymal structures. DBT can reduce summation artifacts compared to full-field digital mammography (FFDM) by acquiring a series of low-dose mammographic images from different angles. Subsequent postprocessing of projection data allows for visualisation of the full breast thickness over multiple slices that can be analysed manually and with the help of computer-aided detection (CAD) systems. Furthermore, a synthetic mammography image can be reconstructed (synthetic 2D) without additional radiation exposure.^1^ Tomosynthesis combined with mammography (FFDM or synthetic 2D) has been shown to improve cancer detection rates in different population-based screening settings^2–11^ and is incorporated in the current European Guidelines.^12^ While higher cancer detection rates are well established, contradictory results remain for recall rates.^13–15^ However, a recently published meta-analysis^16^ suggested fewer recalls when employing DBT including synthetic 2D compared to FFDM alone, as well as higher cancer detection rates among recalls.

On the other hand, studies have demonstrated a substantial increase in reading time, as much as doubling time per study when adding DBT to standard FFDM.^6,17^ This is particularly important in the screening setting, where the number of patients is considerably higher than in a diagnostic assessment setting. Standard DBT protocols usually rely on reconstructed slices with 1 mm thickness, hence, depending on breast thickness, up to a few hundred slices are generated per patient *vs* four standard projections in conventional mammography. In addition to increased reading time, considerably more storage space in picture archiving and communication systems is necessitated.^18^ Above all, however, concerns regarding overall workload for reading radiologists and accompanying fatigue resulting in false assessment ought to be addressed.^19^ With significantly more examinations being read by a radiologist per day, the number of images per DBT is especially impactful in the screening setting. Considering that diagnostic assessment demands a precise characterisation of lesions and includes a larger proportion of pathologic findings, however, the challenges for radiologists in this setting are not to be underestimated either.

Few studies have investigated the issue of optimal slab thickness in DBT with varying recommendations.^20,21^ Since these prior analyses derived their populations mostly from screening centres, the purpose of this investigation was to analyse the interpretation performance and reading time in DBT by comparing standard 1 mm slices *vs* artificial-intelligence-enhanced synthetic thick slabs of 6 mm in a diagnostic assessment setting.

## Methods and materials

This single-centre study was performed after gaining permission from the local institutional review board (IRB number: 20220425 02). The need for additional written informed consent was waived due to the retrospective study design. The Standards for Reporting of Diagnostic Accuracy (STARD) guidelines were adhered to.

### Population

Patients undergoing DBT from September 2020 through December 2021 for further assessment of mammographic findings in a diagnostic setting at a tertiary-care university hospital were included in this retrospective evaluation. Since our institution is no dedicated screening centre, all patients were either symptomatic (20 cases, 19.8%), had a conspicuous finding in a previous examination (42 cases, 41.6%), were evaluated preoperatively for biopsy-proven malignancy (29 cases, 28.7%), or received further assessment during/after cancer therapy (10, 9.9%). The centre assesses 2500 cases per annum and the number of primary breast cancer treatments performed in our hospital in 2021 was approximately 300. Inclusion criteria for this study were defined as presence of one or more lesions with either histopathological correlation or benign result as per one-year consistency, definite benign correlate in ultrasound or pure summation artefact (asymmetry). Exclusion criteria comprised incomplete datasets, lack of follow-up or prior biopsy with clip insertion. Adhering to these standards, a total of 101 patients were eligible for study inclusion. The population consisted of 100 women and one male with a mean age of 59.7 ± 12.1 years. The left breast was examined in 64 cases (57.7%). When stratified by fibroglandular tissue type according to the American College of Radiology classification system, parenchymal density was “a” in 7 patients (6.3%), “b” in 57 patients (51.4%), “c” in 37 patients (33.3%), and “d” in 10 patients (9.0%).

### Imaging

**Figure 1.**
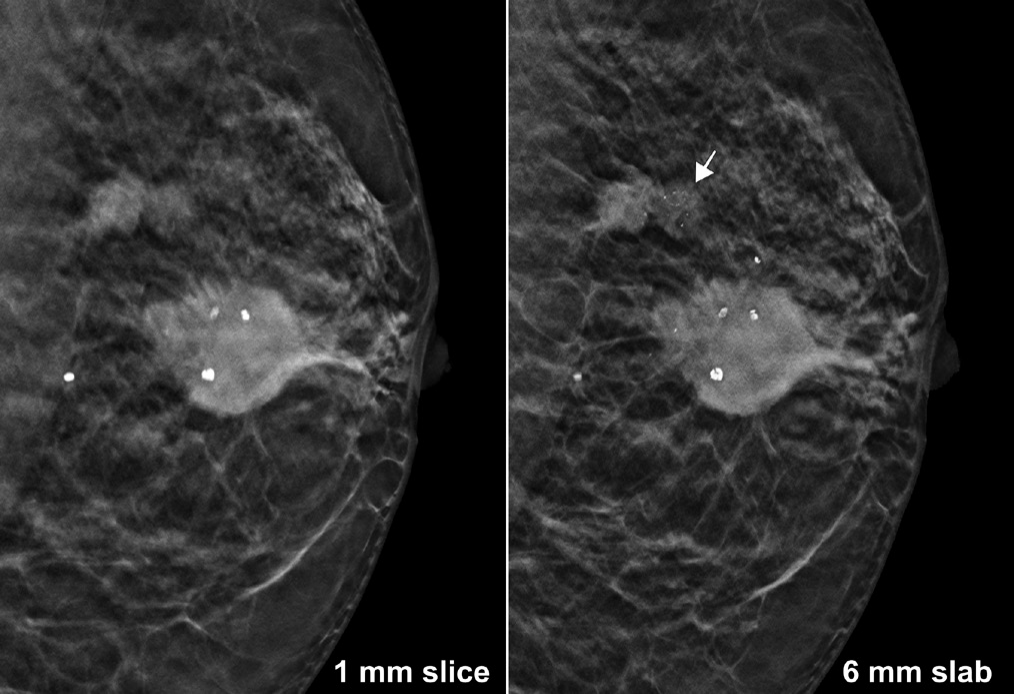
59-year-old female with multifocal invasive breast carcinoma of no special type and associated ductal carcinoma *in situ* in the left upper outer quadrant. The AI-enhanced 6 mm slabs facilitated superior delineation of the associated clustered microcalcifications (arrow) and the spiculated margins of multiple masses as compared to the respective 1 mm slice.

All DBT scans were performed either in mediolateral oblique (MLO: 78 patients; 70.3%), craniocaudal (CC: 21; 18.9%), or lateromedial orientation (LM: 12; 10.8%) with a dedicated mammography system (3Dimensions, Hologic Inc., Marlborough, Massachusetts, USA). Mean compression thickness was calculated at 54.1 ± 14.2 mm for MLO, at 60.0 ± 14.2 mm for CC, and at 47.3 ± 12.8 mm for LM. From each scan, two datasets were reconstructed, consisting either of synthetic 6 mm slabs with 3 mm overlap (“thick slabs”), or standard 1 mm slices (“thin slices”). In order to offset potential blurring and impaired delineation of regions of interest in maximum intensity projections, a vendor-specific algorithm enhanced by artificial intelligence (AI) was employed for generation of thick slabs (3DQuorum, Hologic). This machine-learning algorithm analyses the standard 1 mm slices for various findings such as bright spots, circumscribed densities, or radiating lines and emphasises these in the process of merging the thin slices in order to maintain conspicuity, especially with regard to minute lesions. While a CAD system is also integrated in the software, only the unmarked thick slabs were part of the reader analysis. An example depicting conventional slices *vs* AI-enhanced slabs in a case with several mass lesions and associated clustered microcalcifications representing a multifocal invasive breast carcinoma is provided in Figure 1. Synthetic 6 mm datasets included 22 ± 5 (MLO), 20 ± 5 (CC), and 18 ± 4 (LM) thick slabs, while standard 1 mm stacks comprised of 66 ± 14 (MLO), 60 ± 14 (CC), and 66 ± 12 (LM) thin slices. Effectively, thick slab datasets were reduced by two-thirds of the standard file size.

### Image analysis

Three radiologists with 6 (R1), 4 (R2), and 2 years (R3) of clinical work experience in breast imaging and DBT reviewed all datasets separately, independent from one another, and in randomised order. A 30-day washout period between cases of the same patient was ensured to minimise recall bias. The readers were provided with identical viewing setups on certified diagnostic monitors and a standardised assortment of individual datasets including the FFDM that warranted further assessment via DBT. Prior to commencement of study reading sessions, one author met with the readers and individually reviewed five training cases not included in the study population in order to accustom readers to the study workflow. Readers were blinded to any clinical and histopathological information, as well as to previous imaging studies. Lesions could be characterised as masses, asymmetries, microcalcifications, and architectural distortions. Masses associated with microcalcifications were classified as mass findings. Additional lesions within the same dataset were only evaluated if clearly not associated with the primary findings. For each lesion, readers noted Breast Imaging Reporting and Data System (BIRADS) categories and diagnostic confidence based on an equidistant 5-point-scale (1 = very low confidence, 5 = total confidence). For the calculation of diagnostic accuracy, BIRADS 4 and 5 categories were deemed indicative of malignancy suspicion, whereas BIRADS 1, 2, and 3 categories were considered to represent a primarily non-malignant assessment. As readers were blinded to histopathological information, BIRADS 6 categories could not be assigned for any finding. Furthermore, since all findings were unanimously proven to be either benign or malignant, readers were not allowed to attribute a BIRADS 0 category. Individual reading times, which included interpreting the case and opening/closing the file, were measured manually. Notably, time recordings were started by an external observer when the interpreting radiologist opened a case file and stopped when the radiologist closed the case file in the PACS system. In order to provide consistent measurements and since loading time is negligible on the diagnostic workstations positioned in our department (latency below 2 s), these intervals were not captured separately and the time from file opening to closing was considered representative of the actual interpretation time.

### Data analysis

Data analysis was supported by dedicated software (SPSS Statistics 28, IBM, Armonk, USA). Presentation of non-parametric variables includes absolute numbers and frequencies with median values and interquartile ranges (IQR). Wilcoxon signed rank tests were employed for comparison of suchlike data. With regard to normally distributed parametric data, mean values ± standard deviation were reported and results were compared with paired student’s t-tests. Differences in classification functions of diagnostic accuracy between thick slabs and thin slices were assessed by means of the McNemar test. For interrater reliability, the intraclass correlation coefficient was calculated in a two-way random effects model that analysed the absolute agreement of single measures. An α level of 0.05 was deemed representative of statistical significance.

## Results

Within the study population of 101 individuals, 111 DBT examinations were performed and a total of 115 findings were identified. Of the latter, 65 lesions constituted masses (56.5%), 26 asymmetries (22.6%), 16 microcalcifications (13.9%), and 8 architectural distortions (7.0%). A total of 70 lesions (60.9%) were histopathologically correlated and 56 malignancies (48.7%) were diagnosed. Among these, 42 (75.0%) were invasive carcinomas of no special type (NST), while 7 (12.5%) and 4 (7.1%) lesions were classified as lobular carcinomas and ductal carcinomas *in situ* (DCIS), respectively. The remaining three lesions (5.4%) represented other malignant subtypes. The mean pathological size of malignancies in this study was 30.8 ± 33.7 mm. Accordingly, 59 findings represented benign entities (51.3%), either backed-up by histopathological correlation (14 lesions, 23.7% of benign findings), one-year consistency, benign correlate in ultrasound, or resolved summation artefact (asymmetry). Lesions characteristics within the study sample are summarised in Table 1.

**Table 1.** Study sample

Study sample	
Patients	101
Examinations	111
**Lesion characterisation**	
Overall	115 (100%)
Masses	65 (56.5%)
Asymmetries	26 (22.6%)
Microcalcifications	16 (13.9%)
Architectural distortions	8 (7.0%)
**Malignant lesions**	56 (48.7%)
Carcinoma of no special type	42 (75.0%)
Lobular carcinoma	7 (12.5%)
Ductal carcinoma *in situ*	4 (7.1%)
Other	3 (5.3%)
Size (mean ± standard deviation)	30.8 ± 33.7 mm
**Benign lesions**	59 (51.3%)

### Reading results and diagnostic confidence

Frequency of individual BIRADS categories attributed by each reader for thick slabs and thin slices are presented in Table 2. No significant difference was found between BIRADS categories assigned based on 6 mm *vs* 1 mm datasets (*p* ≥ 0.317). Diagnostic accuracy was comparable for readings of slabs and slices (R1: 87.0% *vs* 87.0%; R2: 86.1% *vs* 87.0%; R3: 80.0% *vs* 84.4%; *p* ≥ 0.125). In 1 mm slices, readers missed one malignant lesion each. In 6-mm synthetic thick slabs, all readers missed the same carcinoma, while R3 did not detect an additional malignancy. Diagnostic sensitivity among readers ranged between 96.43 and 98.21%. A comprehensive display of classification functions of diagnostic accuracy is provided in Table 3. Good interrater reliability was found for both slabs (ICC 0.848; 95% confidence interval 0.800–0.888; *p* < 0.001) and slices (ICC 0.865; 95% confidence interval 0.822–0.901; *p* < 0.001).

**Table 2.** BIRADS categories assigned by each reader for findings in synthetic 6-mm-thick slabs and 1-mm-thin slices are presented as absolute frequencies with percentages in parentheses

Reader	Reader 1		Reader 2		Reader 3	
Dataset	6 mm slabs	1 mm slices	6 mm slabs	1 mm slices	6 mm slabs	1 mm slices
BIRADS 0	–	–	–	–	–	–
BIRADS 1	0	0	0	0	0	0
BIRADS 2	45 (39.13)	45 (39.13)	37 (32.17)	39 (33.91)	39 (33.91)	39 (33.91)
BIRADS 3	1 (0.86)	1 (0.86)	8 (6.96)	7 (6.08)	1 (0.86)	4 (3.48)
BIRADS 4	21 (18.26)	22 (19.13)	28 (24.35)	26 (22.61)	34 (29.57)	30 (26.09)
BIRADS 5	48 (41.74)	47 (40.87)	42 (36.52)	43 (37.39)	41 (35.65)	42 (36.52)
BIRADS 6	–	–	–	–	–	–

**Table 3.** Classification functions were calculated individually per reader for the detection and exclusion of histopathologically-correlated malignant findings

Dataset	6 mm thick slabs	1 mm thin slices	*p* value
**Reader 1**			
Specificity	76.21 [63.41–86.38] (45/59)	76.21 [63.41–86.38] (45/59)	>0.999
Sensitivity	98.21 [90.45–99.95] (55/56)	98.21 [90.45–99.95] (55/56)	
Accuracy	86.96 [79.40–92.51] (100/115)	86.96 [79.40–92.51] (100/115)	
**Reader 2**			
Specificity	74.58 [61.56–85.02] (44/59)	76.21 [63.41–86.38] (45/59)	>0.999
Sensitivity	98.21 [90.45–99.95] (55/56)	98.21 [90.45–99.95] (55/56)	
Accuracy	86.09 [78.39–91.83] (99/115)	86.96 [79.40–92.51] (100/115)	
**Reader 3**			
Specificity	64.41 [50.87–76.45] (38/59)	71.19 [57.92–82.24] (42/59)	0.125
Sensitivity	96.43 [87.69–99.56] (54/56)	98.21 [90.45–99.95] (55/56)	
Accuracy	80.00 [71.52–86.88] (92/115)	84.35 [76.40–90.45] (97/115)	

**Figure 2.**
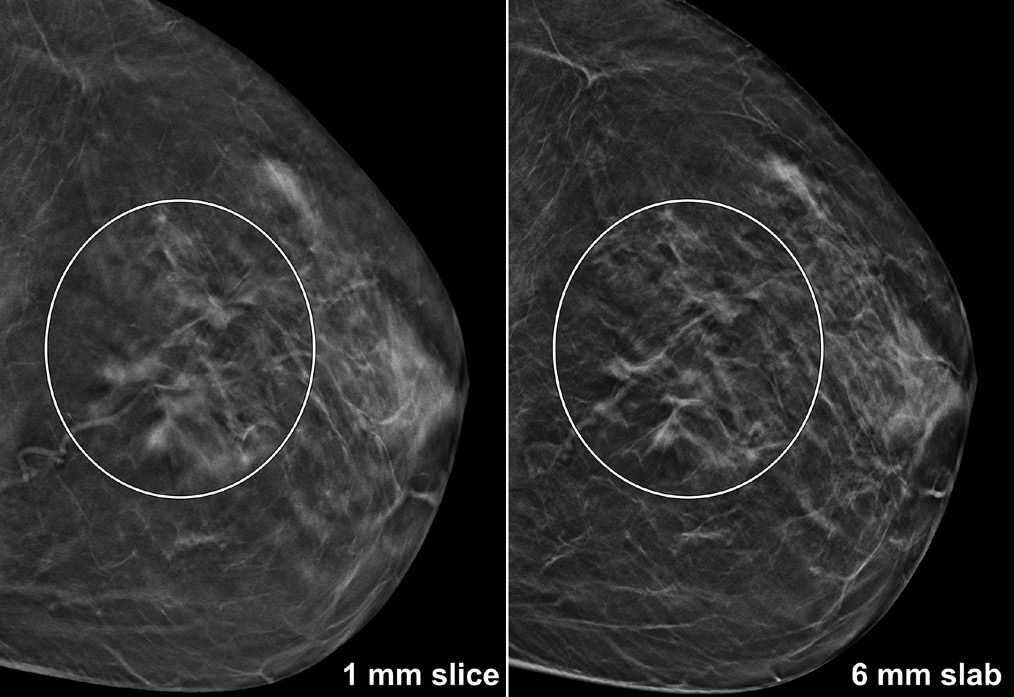
56-year-old female with histologically proven multicentric invasive breast carcinoma of no special type. 6 mm slabs allowed for comprehensive depiction of the architectural distortion’s extent, while simultaneously visualising the associated spiculated masses within the same slab.

Median overall diagnostic confidence of all three readers was at least good for thick slabs (R1: 5 [4-5]; R2: 5 [4-5]; R3: 4 [3-5]) and thin slices (R1: 5 [4-5]; R2: 4 [4-5]; R3: 4 [3-5]). Individual confidence ratings are summarised in Table 4. Only one of three radiologists reported higher confidence levels when interpreting 1 mm slices (R1: *p* = 0.033), while no significant difference was found for the others (*p* ≥ 0.273). Albeit correctly identified by all readers irrespective of section thickness, superior delineation of an architectural distortion in 6 mm datasets may have led to higher confidence ratings in the exemplary case shown in Figure 2.

**Table 4.** Confidence ratings by each reader for findings in synthetic 6-mm-thick slabs and 1-mm-thin slices are presented as absolute frequencies with percentages in parentheses

Reader	Reader 1		Reader 2		Reader 3	
Dataset	6 mm slabs	1 mm slices	6 mm slabs	1 mm slices	6 mm slabs	1 mm slices
Very low confidence	0	0	0	0	4 (3.48)	4 (3.48)
Low confidence	0	0	6 (5.22)	6 (5.22)	11 (9.57)	9 (7.83)
Moderate confidence	6 (5.22)	5 (4.35)	14 (12.17)	15 (13.04)	18 (15.65)	26 (22.61)
High confidence	32 (27.83)	26 (22.61)	32 (27.83)	38 (33.04)	36 (31.30)	32 (27.83)
Complete confidence	77 (66.96)	84 (73.04)	63 (54.78)	56 (48.70)	46 (40.00)	44 (38.26)
**Median**	**5**	**5**	**5**	**4**	**4**	**4**
**IQR**	**4 – 5**	**4 – 5**	**4 – 5**	**4 – 5**	**3 – 5**	**3 – 5**

### Reading time

Mean interpretation time was found to be substantially reduced for 6 mm slabs compared to 1 mm slices. Reading time per tomosynthesis was shortened by 27.4% for R1 (33.5 ± 5.9 *vs* 46.2 ± 5.8 sec; *p* < 0.001), by 24.3% for R2 (49.1 ± 23.5 *vs* 64.8 ± 32.1 sec; *p* < 0.001), and by 41.3% for R3 (39.5 ± 24.7 *vs* 67.2 ± 46.2 sec; *p* < 0.001).

## Discussion

In this retrospective multireader study, we could demonstrate a significant reduction in reading time for synthetic 6 mm slabs enhanced by artificial intelligence *vs* 1 mm slices for digital breast tomosynthesis in a diagnostic setting without a decrease in diagnostic accuracy. All readers stated at least good confidence levels regarding interpretation of the synthetic slabs.

**Figure 3.**
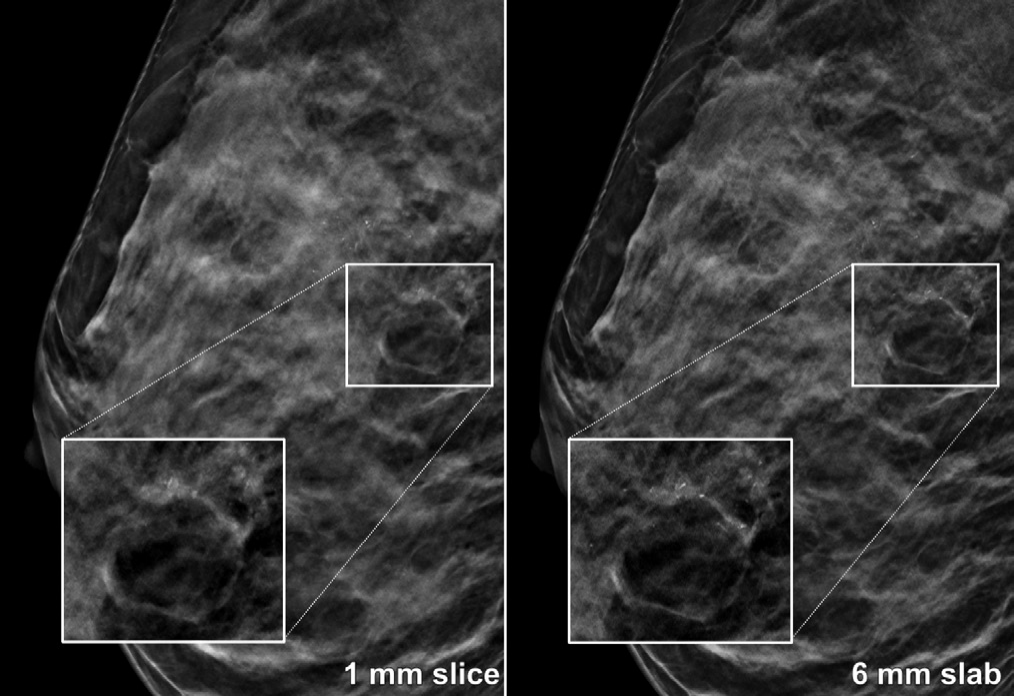
37-year-old female with biopsy-proven invasive breast cancer of no special type and associated ductal carcinoma *in situ*. Reading time of three radiologists was substantially shorter for 6 mm slabs compared to 1 mm slices, presumably due to superior visualisation of regional microcalcifications.

Our findings are in line with the results of Pujara et al., who found reduced reading time in three-quarters of all examined cases when employing a DBT protocol with 6 mm slabs while maintaining diagnostic performance.^21^ Notably, the population of this investigation consisted of asymptomatic females who were referred for breast cancer screening, diagnostic evaluation, or biopsy. Furthermore, different mammography equipment and PACS software/image hangings were used, suggesting a certain degree of transferability of results to other study settings. Figure 3 illustrates a case of multicentric invasive disease and clustered microcalcifications from our population, where the complete extent of the aberration is depicted within one of the 6 mm slabs. Although the lesion was correctly identified by all three readers irrespective of slice thickness, superior delineation in the synthetic slabs and less scrolling may have contributed to the marked reduction in reading time by as much as 41.3% in the case of the most inexperienced reader. The diagnostic sensitivity achieved in our study with the use of 6-mm-thick synthetic slabs was superior compared to the findings of Iotti et al, who employed 10 mm slabs with 5 mm overlap for a 20–30% reduction of interpretation time.^20^ Of note, the authors of this publication relied on a mostly asymptomatic population derived from a screening trial, which was divided into a “specificity set” (12 cancers *vs* 882 negative DBT scans) and a “sensitivity set” (28 cancers *vs* 276 negative DBT scans), resulting in a high overall proportion of negative exams in their population. Despite the different study samples and higher slab thickness used by Iotti et al., the absolute and relative interpretation time reduction were comparable to our study. With that being said, we agree with the deduction of Pujara et al that 6 mm may be the ‘sweet spot’ for slab thickness, since the time saving effect with thicker slabs appears to be negligible compared to the ensuing loss of diagnostic sensitivity. However, we believe that the lesion size must be taken into consideration when designing a thick slab protocol. Since the mean pathologic lesion size in this study was 30.8 ± 33.7 mm and therefore significantly larger than what would be expected in a dedicated screening centre, different specifications regarding slab thickness and overlap may produce superior results in other settings.

Partly in contrast to the results of the present study, Pujara et al reported reader confidence to be inferior for their experimental protocol with 6 mm slabs compared to a standard protocol with 1 mm slices despite their readers’ longstanding experience in breast imaging which ranged from 10 to 30 years.^21^ Possibly, a certain learning or accommodation period for new approaches ought to be considered, especially for users with long-term expertise in the field. Supporting this line of argument, *i.e*. that habituation to the commonly utilised 1 mm slices leads to inferior assessment of the newer thick slab technique, may also be reflected in our findings, as only the most experienced reader reported (slightly) lower confidence levels for reading 6 mm slabs.

Friedewald et al. described an approach to interpreting DBT images by scrolling through the reconstructed stack multiple times and focusing on different compartments of the breast during each pass.^22^ It may be assumed that this technique synergises particularly well with the use of thick slabs due to less images being reviewed each time. In addition to a standardised viewing setup, toggling between prior and current examinations may also decrease the required reading time of radiologists.^23^ A more advanced technique that can reduce interpretation time up to 30% is employment of CAD systems.^24–26^ While the algorithm responsible for AI-enhanced postprocessing of 1 mm slices to create the synthetic thick slabs in our study does provide additional CAD, the results of these analyses were not accessible to the readers in order to maintain comparability of interpretation time and confidence. It remains to be seen whether the combination of CAD systems and AI-enhanced thick slabs brings further benefits compared to each technique on its own. Further investigations are also warranted with direct comparison of manually reconstructed 6 mm slabs as maximum intensity projections *vs* AI-enhanced synthetic 6 mm slabs.

## Limitations

Some limitations inherent to the current study need to be addressed. For one, the retrospective study design cancels all consequences regarding faulty interpretations, possibly further impacting interpretation times in general due to the lack of clinical pressure. Because of the nature of the study, no randomised negative control group was included.The proportions of microcalcifications (13.9%) and DCIS (7.1%) within the group of histopathologically proven malignancies were statistically underrepresented in our dataset and do not reflect the typical frequency in a screening centre, where approximately 25% of cancers are DCIS. Furthermore, the mean pathologic lesion size in our patient sample was considerably larger than the vast majority of findings encountered in a dedicated screening setup. Breast screening programmes such as the one in the UK stipulate minimal standards for breast cancer size detection under 15 mm (concerns more than 55% of both prevalent and incident round cancers). Therefore, limited generalisability of the presented results must be presumed for a dedicated screening setting. Of note, since tomosynthesis is considered to be less useful for assessment of microcalcifications than of masses, additional technical and software developments are required for the display of calcified lesions in DBT in general. Particularly, the current tendency to replace FFDM with synthetic mammograms from DBT examinations emphasises the need for proper solutions in that regard.^27^ Lastly, our study was a single-institution and single-manufacturer investigation, hence the actual benefit of the AI-based reconstruction method cannot be quantified, since no conventional maximum intensity projections were available for comparison. Future studies should aim to replicate findings in larger datasets with additional unenhanced standard 6 mm slabs.

## Conclusions

Employing a reconstruction protocol with artificial-intelligence-enhanced synthetic 6 mm slabs instead of 1 mm slices for digital breast tomosynthesis allowed for substantial interpretation time reduction without a decrease ininterpretation accuracy in the presented diagnostic setting.

## References

[1] Abdullah P, Alabousi M, Ramadan S, Zawawi I, Zawawi M, Bhogadi Y, et al . Synthetic 2D mammography versus standard 2D digital mammography: a diagnostic test accuracy systematic review and meta-analysis. AJR Am J Roentgenol 2021; 217: 314–25. doi: 10.2214/AJR.20.24204 32966115

[2] Heindel W, Weigel S, Gerß J, Hense H-W, Sommer A, Krischke M, et al . Digital breast tomosynthesis plus synthesised mammography versus digital screening mammography for the detection of invasive breast cancer (TOSYMA): a multicentre, open-label, randomised, controlled, superiority trial. Lancet Oncol 2022; 23: 601–11. doi: 10.1016/S1470-2045(22)00194-2 35427470

[3] Alabousi M, Wadera A, Kashif Al-Ghita M, Kashef Al-Ghetaa R, Salameh J-P, Pozdnyakov A, et al . Performance of digital breast tomosynthesis, synthetic mammography, and digital mammography in breast cancer screening: a systematic review and meta-analysis. J Natl Cancer Inst 2021; 113: 680–90. doi: 10.1093/jnci/djaa205 33372954 PMC8168096

[4] Skaane P, Bandos AI, Niklason LT, Sebuødegård S, Østerås BH, Gullien R, et al . Digital mammography versus digital mammography plus tomosynthesis in breast cancer screening: the Oslo tomosynthesis screening trial. Radiology 2019; 291: 23–30. doi: 10.1148/radiol.2019182394 30777808

[5] Hofvind S, Hovda T, Holen ÅS, Lee CI, Albertsen J, Bjørndal H, et al . Digital breast tomosynthesis and synthetic 2D mammography versus digital mammography: evaluation in a population-based screening program. Radiology 2018; 287: 787–94. doi: 10.1148/radiol.2018171361 29494322

[6] Pattacini P, Nitrosi A, Giorgi Rossi P, Iotti V, Ginocchi V, Ravaioli S, et al . Digital mammography versus digital mammography plus tomosynthesis for breast cancer screening: the reggio emilia tomosynthesis randomized trial. Radiology 2018; 288: 375–85. doi: 10.1148/radiol.2018172119 29869961

[7] Zackrisson S, Lång K, Rosso A, Johnson K, Dustler M, Förnvik D, et al . One-view breast tomosynthesis versus two-view mammography in the Malmö breast tomosynthesis screening trial (MBTST): a prospective, population-based, diagnostic accuracy study. Lancet Oncol 2018; 19: 1493–1503. doi: 10.1016/S1470-2045(18)30521-7 30322817

[8] Bernardi D, Macaskill P, Pellegrini M, Valentini M, Fantò C, Ostillio L, et al . Breast cancer screening with tomosynthesis (3D mammography) with acquired or synthetic 2D mammography compared with 2D mammography alone (STORM-2): a population-based prospective study. Lancet Oncol 2016; 17: 1105–13. doi: 10.1016/S1470-2045(16)30101-2 27345635

[9] Conant EF, Beaber EF, Sprague BL, Herschorn SD, Weaver DL, Onega T, et al . Breast cancer screening using tomosynthesis in combination with digital mammography compared to digital mammography alone: a cohort study within the PROSPR Consortium. Breast Cancer Res Treat 2016; 156: 109–16. doi: 10.1007/s10549-016-3695-1 26931450 PMC5536249

[10] Gilbert FJ, Tucker L, Gillan MG, Willsher P, Cooke J, Duncan KA, et al . The Tommy trial: a comparison of tomosynthesis with digital mammography in the UK NHS breast screening programme -- a multicentre retrospective reading study comparing the diagnostic performance of digital breast tomosynthesis and digital mammography with digital mammography alone. Health Technol Assess 2015; 19: i–xxv. doi: 10.3310/hta19040 PMC4781321 25599513

[11] Ciatto S, Houssami N, Bernardi D, Caumo F, Pellegrini M, Brunelli S, et al . Integration of 3D digital mammography with tomosynthesis for population breast-cancer screening (STORM): a prospective comparison study. Lancet Oncol 2013; 14: 583–89. doi: 10.1016/S1470-2045(13)70134-7 23623721

[12] Sardanelli F, Aase HS, Álvarez M, et al . Position paper on screening for breast cancer by the european society of breast imaging (EUSOBI) and 30 national breast radiology bodies from austria, belgium, bosnia and herzegovina bulgaria, croatia, czech republic, denmark, estonia, finland, france, germany, greece, hungary, iceland, ireland, italy, israel, lithuania, moldova, the netherlands, norway, poland, portugal, romania, serbia, slovakia, spain, sweden, switzerland and turkey. Eur Radiol 2017; 27: 2737–43. doi: 10.1007/s00330-016-4612-z 27807699 PMC5486792

[13] Bernardi D, Gentilini MA, De Nisi M, Pellegrini M, Fantò C, Valentini M, et al . Effect of implementing digital breast tomosynthesis (DBT) instead of mammography on population screening outcomes including interval cancer rates: results of the trento DBT pilot evaluation. Breast 2020; 50: 135–40. doi: 10.1016/j.breast.2019.09.012 31607526 PMC7375541

[14] Hofvind S, Sagstad S, Sebuødegård S, Chen Y, Roman M, Lee CI . Interval breast cancer rates and histopathologic tumor characteristics after false-positive findings at mammography in a population-based screening program. Radiology 2018; 287: 58–67. doi: 10.1148/radiol.2017162159 29239711

[15] Houssami N, Hofvind S, Soerensen AL, Robledo KP, Hunter K, Bernardi D, et al . Interval breast cancer rates for digital breast tomosynthesis versus digital mammography population screening: an individual participant data meta-analysis. EClinicalMedicine 2021; 34: : 100804. doi: 10.1016/j.eclinm.2021.100804 33997729 PMC8102709

[16] Heywang-Köbrunner SH, Jänsch A, Hacker A, Weinand S, Vogelmann T . Digital breast tomosynthesis (DBT) plus synthesised two-dimensional mammography (s2d) in breast cancer screening is associated with higher cancer detection and lower recalls compared to digital mammography (DM) alone: results of a systematic review and meta-analysis. Eur Radiol 2022; 32: 2301–12. doi: 10.1007/s00330-021-08308-8 34694451 PMC8921114

[17] Dang PA, Freer PE, Humphrey KL, Halpern EF, Rafferty EA . Addition of tomosynthesis to conventional digital mammography: effect on image interpretation time of screening examinations. Radiology 2014; 270: 49–56. doi: 10.1148/radiol.13130765 24354377

[18] Hardesty LA, Kreidler SM, Glueck DH . Digital breast tomosynthesis utilization in the United States: a survey of physician members of the Society of breast imaging. J Am Coll Radiol 2014; 11: 594–99. doi: 10.1016/j.jacr.2013.11.025 24713501

[19] Tang YZ, Al-Arnawoot A, Alabousi A . The impact of slice thickness on diagnostic accuracy in digital breast tomosynthesis. Can Assoc Radiol J 2022; 73: 535–41. doi: 10.1177/08465371211068200 35193417

[20] Iotti V, Giorgi Rossi P, Nitrosi A, Ravaioli S, Vacondio R, Campari C, et al . Comparing two visualization protocols for tomosynthesis in screening: specificity and sensitivity of slabs versus planes plus slabs. Eur Radiol 2019; 29: 3802–11. doi: 10.1007/s00330-018-5978-x 30737568

[21] Pujara AC, Joe AI, Patterson SK, Neal CH, Noroozian M, Ma T, et al . Digital breast tomosynthesis slab thickness: impact on reader performance and interpretation time. Radiology 2020; 297: 534–42. doi: 10.1148/radiol.2020192805 33021891

[22] Friedewald SM, Bhole S, Wang L, Gupta D . Digital breast tomosynthesis: clinical operations. Journal of Breast Imaging 2019; 1: 122–26. doi: 10.1093/jbi/wbz007 38424919

[23] Drew T, Aizenman AM, Thompson MB, Kovacs MD, Trambert M, Reicher MA, et al . Image toggling saves time in mammography. J Med Imaging (Bellingham) 2016; 3(1): 011003. doi: 10.1117/1.JMI.3.1.011003 26870746 PMC4748143

[24] Balleyguier C, Arfi-Rouche J, Levy L, Toubiana PR, Cohen-Scali F, Toledano AY, et al . Improving digital breast tomosynthesis reading time: a pilot multi-reader, multi-case study using concurrent computer-aided detection (CAD). Eur J Radiol 2017; 97: 83–89. doi: 10.1016/j.ejrad.2017.10.014 29153373

[25] Chae EY, Kim HH, Cha JH, Shin HJ, Choi WJ . Detection and characterization of breast lesions in a selective diagnostic population: diagnostic accuracy study for comparison between one-view digital breast tomosynthesis and two-view full-field digital mammography. Br J Radiol 2016; 89: : 20150743. doi: 10.1259/bjr.20150743 27072391 PMC5258147

[26] Benedikt RA, Boatsman JE, Swann CA, Kirkpatrick AD, Toledano AY . Concurrent computer-aided detection improves reading time of digital breast tomosynthesis and maintains interpretation performance in a multireader multicase study. AJR Am J Roentgenol 2018; 210: 685–94. doi: 10.2214/AJR.17.18185 29064756

[27] Timberg P, Baath M, Andersson I, Mattsson S, Tingberg A, Ruschin M . Visibility of microcalcification clusters and masses in breast tomosynthesis image volumes and digital mammography: a 4AFC human observer study. Med Phys 2012; 39: 2431–37. doi: 10.1118/1.3694105 22559613

